# Supplementary material for: Clear tracks or missed connections? A qualitative study exploring how interest-holder perceptions of purpose shape the implementation and experience of the six-month review for stroke survivors
Source: PLoS One. 2025 Dec 11;20(12):e0339038. doi: 10.1371/journal.pone.0339038 (PMC12697933; doi:10.1371/journal.pone.0339038)
Supplement: S1 Table — (DOCX) [file pone.0339038.s001.docx]

**S1 Table. Comparison table of the three sites**

|  | **Site A** | **Site B** | **Site C** |
| --- | --- | --- | --- |
| **Region** | East of England | South East | North West |
| **Provider Organisation** | Community NHS Trust | Acute NHS Trust | Third-Sector Organisation |
| **Current Staffing Level within 6MR** | 3x Stroke Specialist Nurses (part-time within 6MR service) | 1x Therapist (part-time within 6MR service) | 3x Stroke Co-ordinators (part-time within 6MR service) |
| **Method of Review** | Face-to-face in clinic setting and home visits (including care homes) or telephone appointments | Majority are telephone appointments (face-to-face in special circumstances) | Home visits or telephone appointments (option for virtual) |
| **Position of 6MR within Stroke Pathway** | Embedded within ESD team | Embedded within ESD team | Delivered as part of a third-sector-led stroke support service |
| **Data collection tool** | In-house tool | Adapted GM-SAT | Adapted GM-SAT |
| **Rurality (ONS Rural Urban Classification)** | Mixed rural and urban: Mostly rural town/fringe surrounding urban city and town | Predominantly urban city and town | Mixed urban and rural: Urban city and town with adjacent rural town and fringe areas |

(6MR – Six-Month Review; ESD – Early Supported Discharge, a model of home-based, multidisciplinary rehabilitation that enables early discharge from hospital; ONS – Office for National Statistics; GM-SAT – Greater Manchester Stroke Assessment Tool©; NHS – National Health Service).
