# Supplementary material for: Clear tracks or missed connections? A qualitative study exploring how interest-holder perceptions of purpose shape the implementation and experience of the six-month review for stroke survivors
Source: PLoS One. 2025 Dec 11;20(12):e0339038. doi: 10.1371/journal.pone.0339038 (PMC12697933; doi:10.1371/journal.pone.0339038)
Supplement: S1 File — (DOCX) [file pone.0339038.s002.docx]

**S2 File. Interview schedules for the three interest-holder groups and observation proforma**

**Interview Schedule:** *Service Providers*

| Introduction | Briefly explain research topic and aims for discussion.  Provide information:   - Interview should take approx 60-90 mins - Fine to not answer anything that makes you uncomfortable   **Reaffirm consent and ensure consent documents are signed.**   - Clarify structure and setting of the 6MR service i.e. staffing / location / process. - Ascertain individuals role within service and any hierarchy. |  |
| --- | --- | --- |
| Purpose | - What do you feel is the purpose of the 6MR? - How does the 6MR "fit in" / integrate in to your stroke pathway? |  |
| Contextual Factors from CICI Framework | - How does the local geography (i.e. rurality, transport) influence the ongoing implementation of your service? *Or, do you feel your service would be run differently in a different setting / context?* - How has your service been designed or adapted to best meet the needs of your patients? - Are you aware of any health inequalities that may impact or be relevant for the 6MR? If so, how have services been adapted to account for this? - Has your service been adapted in response to the level of deprivation in the local area? - Do you feel there are any patient groups that your service has difficulty providing the 6MR for? i.e. NH patients - How does SSNAP influence the implementation or adaptation of your 6MR service? - How do national guidelines influence the implementation or adaptation of your 6MR service? - How much of a priority is the 6MR within your region? - How much scope do you have to change/adapt your service in response to need? - How are your connections with other services within the stroke pathway? |  |
| Implementation Factors from CICI Framework | - With regards to the intended purpose of the 6MR, how do you feel the 6MR achieves this? - What do you feel are the important elements of the 6MR? - Thinking back to the initiation of the 6MR service, were there any issues or problems setting the service up? - How do you evaluate your service to ensure it is sustainable? How has the service changed in response to this? - How has the delivery of the 6MR changed over time? - How confident are you that the 6MR is the best way of meeting patients' needs? What other methods do you feel might be more suitable? - How confident are you that you have the required skills to complete the 6MR well? What additional skills do you feel you need? |  |
| Outcomes | - What factors do you feel influence the uptake of the 6MR? - What benefit do you feel your patients get from the 6MR? - Do you feel the current service is acceptable to service users? |  |
| Closing | - Is there anything else you would like to tell me about your service? - Is there anything else you think I should be asking other people?   Thank you for your time |  |

**Interview Schedule:** *Influencers /* *Commissioners / Manager’s / Leaders*

| Introduction | Briefly explain research topic and aims for discussion.  Provide information:   - Interview should take approx 60 mins - Fine to not answer anything that makes you uncomfortable   **Reaffirm consent and ensure consent documents are signed.**   - Ascertain role within stroke pathway and level of authority / control. |  |
| --- | --- | --- |
| Purpose | - What do you feel is the purpose of the 6MR? - How does the 6MR "fit in" / integrate in to your stroke pathway? - How does information you receive from 6MR services help to plan future services? Any examples? |  |
| Contextual Factors from CICI Framework | - How has the 6MR service been commissioned to best meet the needs of the local population? - Are you aware of any health inequalities that may impact or be relevant for the 6MR? If so, how have services been adapted to account for this? - Do you feel the 6MR is value for money in this area? Is the 6MR a financial priority within your area? Why / why not? - How does SSNAP influence any decision-making with regards to the 6MR? - How do national guidelines influence any decision-making with regards to the 6MR? - How much of a priority is the 6MR within your region? |  |
| Implementation Factors from CICI Framework | - With regards to the intended purpose of the 6MR, how do you feel the 6MR achieves this? What do you feel are the important elements of the 6MR? |  |
| Outcomes | - Have you seen value for money in relation to the 6MR? How is this measured? - Do you anticipate / observe cost savings or benefits in other areas of the stroke pathway? |  |
| Closing | - Is there anything else you would like to tell me about 6MRs? - Is there anything else you think I should be asking other people?   Thank you for your time |  |

**Interview Schedule:** *Service Users*

| Introduction | Briefly explain research topic and aims for discussion.  Provide information:   - Interview should take approx 30-45 mins - Fine to move on if don’t feel comfortable answering any questions that are upsetting or triggering - Let me know if you need to take a break   **Reaffirm consent and ensure consent documents are signed.**   - Collect demographic information (age, social history, functional level, carer etc.) - Can you tell me a little about the impact your stroke has had on you? |  |
| --- | --- | --- |
| Purpose | - What do you feel is the purpose of the 6MR? |  |
| Contextual Factors from CICI Framework | - Can you describe any difficulties you had in accessing the 6MR service? - Was the 6MR helpful? Did it cover / meet your needs? - Did you feel able to discuss issues you wanted to discuss at the 6MR? Were there any issues you weren't able to talk about? |  |
| Implementation Factors from CICI Framework | - Tell me about the clinician / person who delivered your 6MR? (Approachable? Knowledgeable? Holistic?) What did they do well / not so well? - What was your understanding about what would happen following the 6MR? Was it clear to you what you needed to do next? Did you have a clear plan? |  |
| Outcomes | - What has changed for you as a result of the 6MR? - What do you feel you need in terms of your longer-term care? |  |
| Closing | - Is there anything else you would like to tell me about your experience? - Is there anything else you think I should be asking other people?   Thank you for your time |  |

| Date |  |
| --- | --- |
| Site |  |
| Method |  |
| Present |  |

**Observation Proforma**

|  | *Consider…* | Notes |
| --- | --- | --- |
| Purpose | *Explanation of purpose.*  *Users understanding of purpose.* |  |
| Context  &  CAS | *Nature of needs and how well are needs met?*  *Adaptations to context i.e. cultural/language, socioeconomic.*  *Carer involvement.*  *Interactions between provider and user.* |  |
| Outcomes | *Outcomes achieved?*  *Were these satisfactory? Any issues left unresolved?*  *Tools/strategies used to achieve outcomes i.e. coaching, behaviour change* |  |
