# Supplementary material for: Clear tracks or missed connections? A qualitative study exploring how interest-holder perceptions of purpose shape the implementation and experience of the six-month review for stroke survivors
Source: PLoS One. 2025 Dec 11;20(12):e0339038. doi: 10.1371/journal.pone.0339038 (PMC12697933; doi:10.1371/journal.pone.0339038)
Supplement: S2 File — (DOCX) [file pone.0339038.s003.docx]

**S3 File. Anonymised participant quotes and observational reflections by theme**

**Participant code key**

- A, B, or C – Site identifier (Site A, B, or C)
- P – Service provider
- I – Service influencer
- U – Service User
- # – Order of interview within that group
- Obs – Observational data

*Example: AP1 = first service provider interviewed at site A.*

**Mind the gap**

[<Files\\Interviews\\AP1>](72399ab3-52d6-439f-9fdc-8a0da8874729) - § 8 references coded [6.32% Coverage]

Reference 1 - 0.11% Coverage

So, I suppose in a nutshell, it’s to identify unmet needs…

Reference 2 - 0.01% Coverage

support

Reference 3 - 0.75% Coverage

errm on a slightly sort of side not I would say also it’s an opportunity for the patients and sometimes their carers to tell their story (chuckle)…

RH: Mm hm

AP1: …which they haven’t had and I think that’s quite a powerful element that I’ve kind of thought about over the years really, it’s the first time somebody’s actually given them time and space to actually talk about what happened to them.

Reference 4 - 1.64% Coverage

AP1: I think they get, they get to sort of errm maybe understand and process that and maybe clarify some of the points because they don’t often, very rarely do they remember the paramedics side of it, very rarely A+E, they start to maybe remember when they hit the ward somebody came and maybe they did this so you can clarify what happened and what their experience was and errm yes, sort of give them that sort of opportunity really, to really understand and feel like they weren’t going mad and I think for the errm people who were with them, which is usually family member, spouse, its more traumatic in a lot of ways because they’re the ones witnessing what happened. So, for them as well to somebody actually acknowledging that it was a really difficult time for them as well. It’s something that has been really helpful, sort of maybe cathartic experience for them.

Reference 5 - 0.47% Coverage

But other times they just, yeah, they just want to kind of go off on their own….

RH: Yeah… tell their own story.

AP1: …and I just allow that really, it’s what’s important to them rather than me kind of going “oh you must fill this in” (laughter).

Reference 6 - 0.77% Coverage

Yeah sure, I suppose if you’re going to be, sort of, with a manager’s hat on there’s data for SSNAP, errm, which is the end of the 6-month SSNAP data gathering and errm, which is obviously is a useful national tool errm and errm and its part of yes, the errm the stroke strategy that every patient should have 6 weeks, 6 months and annual (laughter)….

RH: Yeah.

AP1: ... which ticks those boxes (laughter)…

Reference 7 - 0.84% Coverage

on SystmOne we have a template, 6 months follow up template, quite unfit for purpose but what it does have is onward referrals and signposting…

RH: Yeah.

AP1: …so we’re invariably bad at filling that in so we’re trying to be better so that we can pull off that at some point to see actually where our main areas errm signposting and referrals you know to see where the unmet needs are outside of the stroke services of which we know there are many

Reference 8 - 1.74% Coverage

I was just thinking if I was a patient or a patient’s family member then you know and they’ve been through you know quite an awful experience I think to have that 45 minutes just dedicated to telling you your story and… sometimes it’s reassurance as well that actually you’re doing really well and you’re doing all the right things. You know, “should I be weight-lifting?” … “no, not really” …errm you know “am I ok to swim?” … “yes lovely” you know. So sometimes it’s just that reassurance and confidence building that I think is invaluable and then you’d hope that they wouldn’t… and obviously don’t have the data for that… you’d hope that they wouldn’t be going back to their primary... you know to the GP or whoever else with anxiety or worry which is a lot of you know what we see at 6 months because they haven’t had that opportunity to be reassured and actually what they’re experiencing is all part of their recovery.

[<Files\\Interviews\\AP2>](f6824c29-38f4-4b9f-92dc-9cfc00c3f5b8) - § 7 references coded [2.59% Coverage]

Reference 1 - 0.60% Coverage

but I think it's a good errm time for them to have a a chat with a, I suppose, a more specialist person in stroke, so again a GP is helpful, but they may not have the experience of, of stroke as, as some of us do because we've worked with people for years who've had strokes for perhaps more attuned to picking up some difficulties that they wouldn't get from the GP

Reference 2 - 0.20% Coverage

also time wise we can offer them a nice 45 minute appointment which just wouldn't happen with a GP surgery at the moment.

Reference 3 - 0.29% Coverage

But I think what you were just describing there goes a little bit deeper into errr that more holistic approach isn't it?

AP2: Yeah, that's kind of absolutely what we try and do

Reference 4 - 0.27% Coverage

Some of it is about just telling the patient they're doing really well and they actually like being told I'm getting on well. I think they're quite grateful for that.

Reference 5 - 0.35% Coverage

So I think it's maybe patients I, I would be interested is it because they don't understand why?
What's it for? Why is it gonna benefit me and is that why maybe some people opt out or don't respond to our, our letter.

Reference 6 - 0.24% Coverage

I sometimes I just think they just loved being seen by somebody and somebody giving them a bit of time, but maybe that's OK.
That's a good thing.

Reference 7 - 0.64% Coverage

but nurses for the things they do, which tend to be more fluffy, maybe… I don't have a better word, but yeah, mood based, all those sorts of things, they're harder to prove, the helpfulness of sometimes, or, you know, just offering support and advice. Errm other then just people filling in a feedback form saying “yes, that was helpful.” Errm but I can't… for an outcome measure fits very easily.

[<Files\\Interviews\\AP3>](cb2f1996-65dd-465d-bcdc-9cfc014d6777) - § 8 references coded [5.94% Coverage]

Reference 1 - 0.14% Coverage

It's a bit fluffy, but, but I think it's important that people feel heard

Reference 2 - 1.06% Coverage

So, some people really want the medical thing, they want, “oh, my wife made me come, I need to about…” you know, so you can morph into, okay, we'll do BMI, we'll do temperature err blood pressure, we'll be scientific, we'll look and see what your results are, what your cholesterol is, so we can do that, obviously, but we can also morph into how people feel, errm what's not okay for them. So, errm, if, after that session, there are things that I need to do to follow up, we are not commissioned for it, but I doubt very much if any of us would not do it.

Reference 3 - 0.26% Coverage

I suppose when we first started, we had the comfort of the LoTS trial, which gave us a framework within which to work, to look at areas.

Reference 4 - 0.36% Coverage

And I guess we've morphed the things that the trust asked us to do, that are more medical, because of course, you know, err heart, blood pressure, time, all of those things can be quantified

Reference 5 - 1.27% Coverage

whereas the more kind of a non-, I won't say fluffy, because I feel that’s… I only, I… because, I, I, well, I'm always just interested in people, not particularly what are they doing this or that or the other, but I understand from working in the NHS that they need to quantify what we do, you know…… I don't really give anybody any answers as to… you can’t… is not like a number or a graph, you know, it doesn't, it can't be quantified. And of course, what you're doing now is listening to what we say and what patients say to look at the value of it. And that hasn't ever been enough, the trials, the research, the research is, is very difficult to quantify, isn't it?

Reference 6 - 1.37% Coverage

It doesn't necessarily mean that somebody is more able to go to work or function or this or that, but they're just more rounded people. And I guess because of the hierarchy, you know, we count people by whether you can walk and go back to work as opposed to be at home. But we're not so interested in that because of the hierarchy of health, I suppose.

In my own head, I don't know if there's a hierarchy of health. I always thought emotional well-being is not as valid by statisticians and data people than, you know, the practicalities of let's get people back to work and be a useful human being in our society. I mean, there's total… [inaudible]… but if somebody is disabled in their home, they still want to be okay.

Reference 7 - 0.98% Coverage

AP3:

So, the things that patients say they felt cared for, they felt they've been given time, they felt they've got advice and reassurance, they felt understood, they felt able to talk them through, they felt helped and supported, and they felt more confident. So, errm, if you're looking into the premise of a person feels… just about a guarantee, it's probably enough, six months, they're calmer, they're more understanding, and they're more errm.. let me think of the word, it's all qualitative stuff, you know?

Reference 8 - 0.51% Coverage

So for me, they're just human beings, but I am privileged to have the opportunity to meet with, and if they've got some unmet needs or difficulties that we can look at that they think might be useful for them, then I'm delighted to, to work with them on those things.

[<Files\\Interviews\\BP1>](69201845-61d5-4612-a3dc-f8ef268e4cdf) - § 3 references coded [1.80% Coverage]

Reference 1 - 0.33% Coverage

I'd say a lot of them, there isn't particularly that many actions from it. It is just generally reassurance and a bit of a conversation.

Reference 2 - 0.93% Coverage

I feel like the benefit of it to the patient often is just talking to somebody, to a professional, getting that reassurance. And that's usually enough, to be honest. I think that's what they want. And often they like, you know, we've been very involved with them when they were discharged from hospital and they want to just tell us how they've been getting on. And feed that back.

Reference 3 - 0.54% Coverage

BP1:

I mean, I guess it depends on the patient really, doesn't it? Some of them might have specific things that they want to be addressed. I think, as I said before, the main theme that comes out is this fatigue thing.

[<Files\\Interviews\\BP2>](1c13c4cd-b0df-4a22-85dc-f8ef29fd367f) - § 3 references coded [0.85% Coverage]

Reference 1 - 0.32% Coverage

It probably does feel a little bit like a tick box in some circumstances that we're just doing it because we're told that it should be done.

Reference 2 - 0.36% Coverage

BP2:

And I suppose I would turn that around to say none of us have really asked the patients of what they want, as in what they're expecting out of them.

Reference 3 - 0.17% Coverage

it's sort of giving them that reassurance that that is part and parcel

[<Files\\Interviews\\BP3>](be095d8a-f764-4b5a-84dc-f8ef2b96ce67) - § 2 references coded [0.82% Coverage]

Reference 1 - 0.37% Coverage

Obviously, I explained to them at the beginning it might not be appropriate for… you know, all the answers.

Some might not be appropriate for them, but we have to ask them all.

Reference 2 - 0.45% Coverage

I often ask that. But why are they accepting a six month period if they've got no issues? And again, I think it's that reassurance and that somebody is listening to them and somebody they have that ongoing support.

[<Files\\Interviews\\CP1>](d4ded1b9-d267-4dff-a7dd-186e6863d789) - § 5 references coded [1.72% Coverage]

Reference 1 - 0.25% Coverage

ultimately, it's about offering a personalised service where that person is at the centre of whatever support we give. So, we like it to be led by them.

Reference 2 - 0.31% Coverage

You know, there's that personal element that, you know, we as humans enjoy, don't we? And it just makes it easier then to receive support. And I don't know, be proactive as a stroke survivor.

Reference 3 - 0.41% Coverage

And to be honest, the face-to-face ones, you know, you can't take away from those, can you, and the valuable, how valuable they are when you actually can see somebody, and you know, it's easier to gauge if they're managing okay and they're coping okay

Reference 4 - 0.25% Coverage

I think that it would be just connecting with the person, identifying unmet needs, providing that support, and just improving outcomes really, ultimately.

Reference 5 - 0.49% Coverage

That's the rewarding part when you've done the review and you've had the input with the person and then you do a follow-up and you can really see the difference it's made. It's helped the person.

So, I think it's just keeping everybody in mind and making it still as person-centered as you can.

[<Files\\Interviews\\CP2>](501f46c8-6a7c-4499-87dd-186e69fb4063) - § 7 references coded [2.36% Coverage]

Reference 1 - 0.23% Coverage

primarily to give stroke survivors and their carers a voice. It's person-centred. So, it starts from where the person is at.

Reference 2 - 0.33% Coverage

And they just want to talk to somebody. So, I had a gentleman the other day who was absolutely lovely, but he was telling me his life story. It's nothing to do with the stroke.

Reference 3 - 0.31% Coverage

but the starting point for the six month review is always the same, which is, you know, what's on your mind at the moment?

What's, what's your biggest concern?

Reference 4 - 0.40% Coverage

Start off by saying what's important to you and then through discussion, identifying other needs they may not have realised were connected to stroke, such as fatigue and providing information and advice around this,

Reference 5 - 0.30% Coverage

it's essential that it's person centred, identifying the needs that they have now, which are likely to be ongoing or unresolved and supporting them with that.

Reference 6 - 0.18% Coverage

So they feel listened to, an opportunity to have their voices heard.

They have an advocate.

Reference 7 - 0.61% Coverage

But as I say, the beauty of the six month review is that it will flag up things more often than not that they are not even aware of that they've just kind of got used to adjusted to, but things that could be improved like fatigue or, you know, or like, um, um, being isolated or whatever, you know, a number of things really.

[<Files\\Observations\\Obs.AP1>](e037026c-6916-4e99-a4dc-bc57b0ac8983) - § 3 references coded [2.83% Coverage]

Reference 1 - 0.90% Coverage

| Purpose | *Explanation of purpose.*  *Users understanding of purpose.* | **To catch-up with how things have been. Check if any worries or concerns.**  Patient immediately into problems (fatigue). Patient seems to be well-informed of the plan for the 6MR via the pre-review information they received. Came with a list of issues they would like to discuss. |
| --- | --- | --- |

Reference 2 - 0.80% Coverage

| Purpose | *Explanation of purpose.*  *Users understanding of purpose.* | **Any worries or concerns? Find out how the patient is getting on.** |
| --- | --- | --- |

Reference 3 - 1.14% Coverage

| Purpose | *Explanation of purpose.*  *Users understanding of purpose.* | **Opportunity to check how things are going. Opportunity for patient to ask anything they want.**  Patient had not used priming tool checklist at home therefore went through this with patient during appointment. AP1 probing further on small mentions of things i.e. anxiety. AP1 appears keen to use this time to cover all necessary things. |
| --- | --- | --- |

[<Files\\Observations\\Obs.AP2>](41fd7182-554f-4da9-a5dc-bc57b0fa0fa5) - § 3 references coded [3.12% Coverage]

Reference 1 - 0.57% Coverage

| Purpose | *Explanation of purpose.*  *Users understanding of purpose.* | ***Residential home***  **Find out how patient is getting on.**  Patient had co-morbidity of dementia. Pre-info sent but patient did not appear to be expecting appointment nor had chance to think about it.  *? if information communicated with patient or evenly alerted to appointment prior. ? if anyone in care home had considered the priming tool.* |
| --- | --- | --- |

Reference 2 - 0.91% Coverage

| Purpose | *Explanation of purpose.*  *Users understanding of purpose.* | **To understand how patient is doing following his stroke.**  Patient had live in carer who seemed to organize all patient’s paperwork and appointments etc. Had gone through the priming tool with patient beforehand. Patient was very laid-back and happy to |
| --- | --- | --- |

Reference 3 - 1.65% Coverage

| Purpose | *Explanation of purpose.*  *Users understanding of purpose.* | ***Nursing home***  **Left open, patient given chance to lead on any topic even if unrealistic i.e. would like to walk again.**  Paperwork completed with NoK (long-term friend) who was present. |
| --- | --- | --- |

**Just passing through?**

[<Files\\Interviews\\AP1>](72399ab3-52d6-439f-9fdc-8a0da8874729) - § 2 references coded [0.13% Coverage]

Reference 1 - 0.07% Coverage

and to then sort of signpost referrals

Reference 2 - 0.06% Coverage

provide education as it comes up

[<Files\\Interviews\\AP2>](f6824c29-38f4-4b9f-92dc-9cfc00c3f5b8) - § 2 references coded [2.30% Coverage]

Reference 1 - 1.96% Coverage

So I think the purpose of it is lifestyle, secondary prevention. So it's a good opportunity to errm look at risk factors and trying to prevent another stroke. Errm I think it's a good opportunity to look for any ongoing difficulties.
So things like ongoing pain or particularly in care homes like contractures or tone problems, that type of thing. Errm I think because I think they picked, I, I would assume they picked six months because it's when recovery of a stroke starts to plateau a bit. Errm and I think it's a good opportunity to have those conversations with patients, to be honest about recovery errm and that have the conversation that things may well slow down now. So people have an understanding of that, that they're recovery can still carry on, but it might just be more slowly. So you're kind of trying to help with their motivation in that sense.
Errm often I find by six months people have, “Are you still doing your exercise and things?” you know “I'm not doing those anymore”, and actually it's it's a good chance to have those conversations about actually this is, you know, this should be a lifelong thing you need to carry on doing these.
They’re always gonna be helpful to you.

Reference 2 - 0.34% Coverage

Errm I guess in some ways it might be to take a bit of pressure off the GP surgery, although often inevitably sometimes because of the six months a lot, we end up referring them to see the GP for certain things,

[<Files\\Interviews\\AP3>](cb2f1996-65dd-465d-bcdc-9cfc014d6777) - § 1 reference coded [0.51% Coverage]

Reference 1 - 0.51% Coverage

So for me, they're just human beings, but I am privileged to have the opportunity to meet with, and if they've got some unmet needs or difficulties that we can look at that they think might be useful for them, then I'm delighted to, to work with them on those things.

[<Files\\Interviews\\BP1>](69201845-61d5-4612-a3dc-f8ef268e4cdf) - § 3 references coded [1.50% Coverage]

Reference 1 - 0.40% Coverage

I mean, it's mainly a review to see how they're getting on six months down the line. And sort of signpost or ensure they've got the support they need at that point.

Reference 2 - 0.62% Coverage

I think we do feel a bit like sometimes that they, you know, in that sort of six month review situation, you're quite limited in what you can do. So, I don't know. I think sometimes the patients want you to actually do something for them, but you can't.

Reference 3 - 0.48% Coverage

And like I was saying earlier, there isn't those neuro specific services in our area to refer on to. It does feel a bit like, I don't know, is there much I can really do for you in this situation?

[<Files\\Interviews\\BP2>](1c13c4cd-b0df-4a22-85dc-f8ef29fd367f) - § 3 references coded [0.89% Coverage]

Reference 1 - 0.31% Coverage

from a clinical perspective is to see how the patients are doing and whether they need further support at that six-month point of view.

Reference 2 - 0.46% Coverage

I think the majority of patients that are left into that still have some questions and therefore it's important to try and have a specialist person help to support them at that point to give them that.

Reference 3 - 0.11% Coverage

Yeah, that's the final support at six months now.

[<Files\\Interviews\\CP1>](d4ded1b9-d267-4dff-a7dd-186e6863d789) - § 7 references coded [2.80% Coverage]

Reference 1 - 0.31% Coverage

what support we can put in place, letting them know what possible outcomes we might be able to help with, whether that's increased independence, reassurance, just feeling supported, really

Reference 2 - 0.31% Coverage

So, hearing about us in more detail and what we can do. You know, at the end of the day, we're there for them then. Although our service is only for 12 months, it's not written in stone.

Reference 3 - 0.21% Coverage

We try and empower them to, you know, help themselves forwards and move forwards as opposed to just doing everything for them.

Reference 4 - 0.34% Coverage

so it could be that they need to have more information around their stroke risk factors to help them manage any future stroke risk and of course that you know runs into secondary stroke prevention doesn't it

Reference 5 - 0.90% Coverage

we have, you know, that information for most identified stroke risks and then that translates to, you know, what we can do to reduce that risk, what healthcare professionals involvement is, so it looks at the medication side of it as well. But ultimately it's there to try and empower a person to know enough to make their own informed decisions and that's all we can do, isn't it? I mean, if someone chooses to smoke, as long as they are aware of the risks and things, that's their decision, but they're not doing it without being aware of that.

Reference 6 - 0.25% Coverage

I think that it would be just connecting with the person, identifying unmet needs, providing that support, and just improving outcomes really, ultimately.

Reference 7 - 0.49% Coverage

That's the rewarding part when you've done the review and you've had the input with the person and then you do a follow-up and you can really see the difference it's made. It's helped the person.

So, I think it's just keeping everybody in mind and making it still as person-centered as you can.

[<Files\\Interviews\\CP2>](501f46c8-6a7c-4499-87dd-186e69fb4063) - § 9 references coded [3.25% Coverage]

Reference 1 - 0.43% Coverage

the purpose is to identify the ongoing issues for stroke survivors. So, as I said before, by six months, some issues will have resolved, others will be ongoing, and also identify anything that has been missed or needs following up.

Reference 2 - 0.67% Coverage

It enables us to have conversations around secondary prevention.

One thing that we do, so when we do an in-person six-month review, we take the person's blood pressure. We can, so again, talk about the importance of that, about monitoring that regularly. We can advise them and reassure them, but also to have open conversations and say things how it is.

Reference 3 - 0.41% Coverage

And for the carer, you know, I know that you're finding it difficult. You'd like to connect with some other people. Would you like a referral to the carer's hub?

See how they can support you? Yes. Okay, that's great.

Reference 4 - 0.51% Coverage

it does need to be person centred, that is critical, identifying the needs that the person has now and which are likely to be ongoing or not resolved, such as pain management, mobility, et cetera. Seeing what we can do to support the stroke survivor with these challenges.

Reference 5 - 0.40% Coverage

Start off by saying what's important to you and then through discussion, identifying other needs they may not have realised were connected to stroke, such as fatigue and providing information and advice around this,

Reference 6 - 0.21% Coverage

providing practical and emotional support, helping them to understand their stroke and how to avoid a recurrence.

Reference 7 - 0.17% Coverage

And the second one is helping them to understand their stroke and how to avoid a recurrence.

Reference 8 - 0.15% Coverage

And if I put a third one in there, practical and emotional support (laughing).

Reference 9 - 0.28% Coverage

so they can talk to someone outside the family, not emotionally involved, able to give information, advice and support and challenge if appropriate.

[<Files\\Observations\\Obs.BP1>](e1f35a7f-46c6-46f4-b7dd-0245f067fc74) - § 2 references coded [6.71% Coverage]

Reference 1 - 5.03% Coverage

| Context  &  CAS | *Nature of needs and how well are needs met?* | **Patient reports primary issues around memory and worry. *BP1* directed patient back to cognitive rehab patient received during ESD and socialising advice. Unable to pick apart specific cause of issue and appeared reluctant to unpick this as would have left her with an inability to refer onwards if an issue is found.**  Increased alcohol reported -> advised on correct amount but little discussion around cutting down.  Patient monitors own BP daily – another example of having a BP machine at home. |
| --- | --- | --- |

Reference 2 - 1.68% Coverage

| Context  &  CAS | *Nature of needs and how well are needs met?* | Primary concern was that his foot was swollen which was impacting on walking. **As 6MR done over the phone there was no opportunity to assess therefore was advised to seek help from GP.**  Checked through secondary prevention risk factors as part of checklist.  Patient reported ongoing balance issues (again, *BP1* unable to assess extent of issues). |
| --- | --- | --- |

**Charting the course**

[<Files\\Interviews\\AI2>](6bc40a10-a652-4245-addc-f73772c7dc26) - § 6 references coded [3.06% Coverage]

Reference 1 - 0.37% Coverage

So I think it is part of, it's a tool to help us understand, collect data because we use SSNAP, but really begin to pull in a much more coordinated, integrated rehabilitation offer.

Reference 2 - 0.74% Coverage

It's almost like we have to try and compare apples with apples. And so I think it is, it's an attempt to, in a very complex, multifactorial delivery, complex intervention. It's, you know, a way of collecting data at a single time point that tries to give consensus and understanding of that fragmentation of what happens, but how much needs still exist for people.

Reference 3 - 0.41% Coverage

But fundamentally, to answer your question in a nutshell, I think it's about trying to get consistency of understanding what that looks like to give us data to drive improvement further, in a nutshell.

Reference 4 - 0.53% Coverage

So, they're beginning to say, this is not acceptable along your pathway, and therefore, then you can lever into transformation.

So, it's a bit of a convoluted answer, but it's about how we influence, and we do use the data, and by keeping the focus on that.

Reference 5 - 0.44% Coverage

There is also another level around, you know, can you make a, can you make a cost-effective argument by collecting your EQ5D that links into your QALYs to then say to the system, this is what you've got to do then.

Reference 6 - 0.57% Coverage

So there is something about understanding those broader measurements around, you know, modified Rankin, around EQ5D-5L, you know, how can we then begin to construct that argument on a financial outcome level that supports health and social care to deliver what it needs to deliver?

[<Files\\Interviews\\BI1>](4684939a-df95-4033-87dc-ff2599f5a74c) - § 1 reference coded [0.34% Coverage]

Reference 1 - 0.34% Coverage

So, there was one of the priorities right back at the beginning was to look at standardising data and looking at what data could do,

[<Files\\Interviews\\BI2>](1f886843-ede4-4848-a1dc-ff259ec48af8) - § 1 reference coded [0.50% Coverage]

Reference 1 - 0.50% Coverage

what data do we have that we can use to compare and understand what's going on in our patch? And the first thing that we said was that, you know, we appreciate SSNAP has its limitations.

[<Files\\Interviews\\BI3>](e44630a0-c691-47f7-a5dd-57d559a3c606) - § 7 references coded [7.94% Coverage]

Reference 1 - 1.62% Coverage

I completely agree that I think the stroke association is around looking at how the person's doing socially, and I've always been interested in to understand how many of those patients are referred back into primary care, potentially. Is that helping primary care to deliver that, or is it just overloading an already very busy service? We hear from patients all the time that they can't get an appointment with their GP, and if they're talking about things that I'm struggling to get back into this, there's, you know, they put on a very long list of things.

Reference 2 - 0.76% Coverage

I think nurses can deliver all of that, if I'm honest. I think they can deliver the medical part, the cardiovascular risks, assess for mood, and refer appropriately to the area of most need for that patient, as opposed to just referring back to, say, primary care.

Reference 3 - 0.29% Coverage

I hope that it reduces primary care burden in the fact that we're offering the ability to speak to.

Reference 4 - 1.75% Coverage

We're getting people back, hopefully, to work, so from an economic value, but I think many of these people, questions that patients have, if they didn't have that six-month review, they would take that potentially to their GP, or they would be then referred back into the stroke team for a further review, so we're also protecting our medical colleagues from that as well, and so the amount of advice and guidance that we get from a daily basis from primary care in different sectors, so whether it be pharmacy, physio, GP, that we're also hopefully stemming that level of work that's coming down as well.

Reference 5 - 0.44% Coverage

BI3:

I think hopefully it helps again support GPs that are going out to those nursing homes that you've you know you've looked at those sorts of things.

Reference 6 - 1.72% Coverage

No, I do think that we could do better in it being value for money, in the fact of, if we're asking the Stroke Association to deliver, understanding that they're not then impacting other services.

I think there needs to be a level of direct referral to places and not, and an awareness of what's available in primary care and how you can refer your patient to that. As opposed to, yeah, I'm going to listen to you and that's really important and I'm going to talk about what the Stroke Association can deliver, but then potentially I'm going to refer you to somebody else to deliver the rest.

Reference 7 - 1.36% Coverage

And looking at the types of stroke and how that can then help guide us to what services are then... we need to commission in the future. So if you are having, so there's an increased amount of intracerebral hemorrhages and this was their main concern. Then how can we, if it's going to continue to increase, what are we going to deliver in the next few years?

So yeah, those sorts of things within the six month reviews I think would be helpful for forward planning.

[<Files\\Interviews\\CI1>](c806e5f0-d18d-49e5-81dd-5fb0ce3245d5) - § 6 references coded [3.41% Coverage]

Reference 1 - 0.70% Coverage

we look at what the data is telling us about, what the needs of stroke survivors are at that time, and try and use that to say, okay, well, there's clear patterns here, but for example, fatigue seems to be a real issue at six months. Can we look at what that's telling us and what services we therefore might need to provide and when?

Reference 2 - 0.28% Coverage

what's that data telling us about the needs of stroke survivors? And are the services there then at the right point in their journey?

Reference 3 - 0.56% Coverage

But there's also an ICB level that we influence to make sure that stroke's a priority there, because if they don't believe stroke's a priority, they won't put aside the money it needs to be able to provide the services in the first place. So I influence there as well.

Reference 4 - 0.45% Coverage

I think without a doubt the NHS and commissioners naturally prioritise the acute end of the pathway. So it can be a real battle, but the more data we have and the more lived experience we have, that helps with that.

Reference 5 - 0.85% Coverage

So I do think it is really helpful to be able to collect that data and then influence for better services for people, because ultimately if we are assessing people and we're finding that they need things but they're not available in the community, then we still can't meet the needs of that person. So that's a secondary element through the data collection that we can see the trends and the needs of people.

Reference 6 - 0.57% Coverage

I guess the other one that I didn't touch on there is one for me that is really useful, and I don't think we do this well at the minute because we're perhaps not collecting the right data, but a potential of it is to look back at what difference initial interventions made.

**Getting things back on track**

[<Files\\Interviews\\AI1>](ccfd8395-3c1c-418a-aadc-b632c1b4fb57) - § 5 references coded [1.81% Coverage]

Reference 1 - 0.32% Coverage

making sure that people who had been through the system had a chance to assess their progress, see whether they had any ongoing needs

Reference 2 - 0.23% Coverage

potentially signpost them into other services at that stage if they'd fallen through the net.

Reference 3 - 0.34% Coverage

I think it gives me reassurance that a patient that I am going to be discharging at some stage will be seen by an expert clinician in stroke.

Reference 4 - 0.29% Coverage

from a lifestyle perspective and kind of secondary prevention perspective, I see that as a very, very key intervention

Reference 5 - 0.63% Coverage

What's their emotional, their mental status? All the things that are hidden, like social phobia, like emotionalism and emotional lability, like the sexual side of things, you know, a lot of people wouldn't talk about that during the first period of a stroke.

[<Files\\Interviews\\AI2>](6bc40a10-a652-4245-addc-f73772c7dc26) - § 2 references coded [0.63% Coverage]

Reference 1 - 0.42% Coverage

whatever we're delivering on stroke pathway it's not enough and I think in my head it's about how we really understand people after stroke, what outcomes and what's still part of their needs at six months.

Reference 2 - 0.21% Coverage

But I think there's something about, there is, as I said, understanding somebody's needs at six months.

[<Files\\Interviews\\BI1>](4684939a-df95-4033-87dc-ff2599f5a74c) - § 7 references coded [3.79% Coverage]

Reference 1 - 0.24% Coverage

I think it's an opportunity to take a very holistic view of the patient's needs at six months.

Reference 2 - 0.43% Coverage

So I'm not sure exactly when the best time of that is, but I think it's more to just look at identifying needs, themes, signposting, and support to those individuals.

Reference 3 - 1.10% Coverage

So identifying, you know, the, um, you know, the needs, um, what support, how people can access that, because I think, I think the way often this works, it's not actually that individual can solve or do all of those things, you know, or take that back or, or access, but it's about again, coordinating services or signposting services that that person can access to hopefully meet the needs or those issues that are existing.

Reference 4 - 0.49% Coverage

But I suppose then that would be probably, it could be a timely point, if that was around six months, that they met with a key worker to then discuss all of those things and then signposting.

Reference 5 - 0.46% Coverage

BI1:

I suppose I, yeah, I suppose it's to understand the needs of the patient at that point. And if any of their needs are unmet, or they need support accessing help for that.

Reference 6 - 0.42% Coverage

You know, so how is, you know, is the secondary prevention sort of advice appropriate? Are they still following that up? Has that needed to be changed or reviewed?

Reference 7 - 0.65% Coverage

So I suppose it's identifying, you know, what, what are they, their needs at that point? And have they changed? Things that may not have needed support with on leaving hospital, they may need now support with, you know, social needs may have changed.

[<Files\\Interviews\\BI2>](1f886843-ede4-4848-a1dc-ff259ec48af8) - § 3 references coded [3.14% Coverage]

Reference 1 - 1.38% Coverage

I think it's around making sure the patients who've been gone through stroke services have an opportunity to sort of sense check, check that they are aware of secondary prevention to prevent them having another stroke and also that they've had every bit of help that they might need because sometimes, you know, because the six months reviews are for everybody who's had a stroke, some people have had a huge amount of input and be very well linked in with services and some people will have had very little input.

Reference 2 - 0.76% Coverage

So, it's just to make sure that everybody has access to a review, making sure that they are checking their medication, checking that's been, you know, that's been optimized and checking that they are aware of, you know, signposting to, you know, to additional things in the community.

Reference 3 - 1.00% Coverage

it feels like a safety net at a time where they might be better able to take that information on board because people, you know, for us, we know that patients have a six-week review, six to eight-week review and then often they have a lot of inputs really early on in the journey but then they aren't always in the best place to actually take that information on board.

[<Files\\Interviews\\BI3>](e44630a0-c691-47f7-a5dd-57d559a3c606) - § 10 references coded [7.50% Coverage]

Reference 1 - 0.29% Coverage

I think it's a chance to see how the patient is at six months and their recovery from their stroke,

Reference 2 - 0.15% Coverage

identifying the support that they need at that time

Reference 3 - 0.40% Coverage

but also the ability to go through their cardiovascular risks and their secondary prevention and discuss in more detail lifestyle choices.

Reference 4 - 0.47% Coverage

It offers the ability to be able to make onward referral for things like exercise plans, getting people back into socialising, group therapy and things like that

Reference 5 - 0.40% Coverage

as well as any questions that the person has and being able to ask that of a stroke specialist. I'm a big advocate for nurse-led services.

Reference 6 - 2.29% Coverage

I feel that the nurse offers a holistic review of the patient, can identify any concerns there with medication. We know through research that about six months, patients go, oh, secondary prevention medication, it's not for me, I'm going to stop it. It's a good chance at that opportunity to reinforce why you're on this medication and any challenges that they've had as well with that, so any adverse reactions.

Establish a routine around blood pressure management, taking their pulse, all those sort of things. At a point where I feel that the patient is, that they've understood how the stroke has affected them, the challenges that they're having, and they're able to, I think, really voice that for us to be able to think what their needs are and how we might be able to address them.

Reference 7 - 0.71% Coverage

But then there's this gap there around, well, lifestyle choices, getting back to exercise. I'm going to make the referral to the Leisure Centre for you to be able to have that, the Stroke Association, exercise groups, have you linked in with this?

Reference 8 - 1.51% Coverage

I think the nurses will holistically think about how this person's recovered and how I'm going to engage them back in everyday life, as well as thinking about supporting them with their mood, adaptation, and having the ability to be able to do that, and perhaps the time as well. So I know the therapists within the ESD team are also very good at doing that, so I think six-month reviews really doesn't fit with the medical world, and very much fits with nursing and therapy, to be able to support the patient at that time.

Reference 9 - 0.69% Coverage

We've reinforced medication, so the person hopefully isn't stockpiling medication and not taking it. We've got good concordance with preventing further stroke by supporting that, so hopefully preventing disability moving forward with that

Reference 10 - 0.59% Coverage

So I would say the medication, taking the medication, addressing the cardiovascular risks and talking them through how they can do that. Their mood and access into everyday life. And then planning forward.

[<Files\\Interviews\\CI1>](c806e5f0-d18d-49e5-81dd-5fb0ce3245d5) - § 7 references coded [3.60% Coverage]

Reference 1 - 0.47% Coverage

a point in time at which we can assess where somebody is and what's important to them at that point and what their needs are and put in plan how we're going to meet those needs, what the support that's needed to meet those.

Reference 2 - 1.31% Coverage

measuring the cow doesn't make it any fatter. So if we just review people, that doesn't help them. So the review itself is, for me, the most important thing about it is that it enables us to continue to meet the changing needs of stroke survivors and their carers, and at that really important time where needs are changing and where services are more formal, NHS services are dropping away, it gives us that opportunity. So I think even if we didn't collect the data, even if we didn't do anything else, that fundamentally is the most important part of them to be able to assess and put in place the plan of support afterwards.

Reference 3 - 0.69% Coverage

there was really clear differences in the data between the therapy teams and our teams. So understandably, our staff picked up far more social issues, far more issues around sex and relationships, issues around connection, and the therapists were far more therapy-related and more of a medical model of issues that they picked up.

Reference 4 - 0.08% Coverage

I think that has to be person-centred.

Reference 5 - 0.17% Coverage

I think where we wanted to say is, what is important to that person at the time?

Reference 6 - 0.40% Coverage

I do think the secondary prevention is an important area in relation to medication management, blood pressure and AF particularly. And then are there lifestyle choices that you want to go into?

Reference 7 - 0.48% Coverage

And I think being able to focus in on what's important to you and move away from, we just need to ask people 35 questions to make sure we've covered this whole assessment, to what's important for you right now is a positive shift.

**‘Tickets please!’**

[<Files\\Interviews\\AU1>](d9bf60e7-662b-41bf-b6dc-a0fab0b5c164) - § 1 reference coded [0.42% Coverage]

Reference 1 - 0.42% Coverage

It did... I just read it roughly... it just said any issues at all relating to it as to how I read it.

[<Files\\Interviews\\AU2>](d183ecfd-45af-43ea-84dc-a4bdfccbbdcf) - § 2 references coded [3.46% Coverage]

Reference 1 - 1.05% Coverage

Yeah, yeah, yeah, yeah, yeah, yeah. Yeah, it was just to see how I was, errm understanding if there's any issues, whatever. And, yeah, yeah, everything… doing my heart monitor, blood pressure, asking initial things, if I'm okay, or how I progressed as well.

Reference 2 - 2.42% Coverage

AU2:

No, I was fine, she explained everything, she asked me about tablets, and various other things, what blood pressure, and how the stroke as well, asked about, about what happened if I get another stroke, what I'd be… and she sort of explained to me, you may get it AU2, you may not, she sort of asked me about alcohol as well, and things like that, so yeah, she said, try minimum alcohol, because of the blood, just do your blood pressure, the blood pressure's okay, but I bought a, I actually bought a blood pressure monitor myself, and the blood is all okay, but just keep an eye on it.

[<Files\\Interviews\\AU3>](3770e238-8c1a-46b6-94dc-afe6a72c03c5) - § 3 references coded [3.40% Coverage]

Reference 1 - 0.85% Coverage

AU3:

That's for future reference, you know, for other people so they'd get, erm, so they know which way to go when you have a stroke, you know. Erm, because I'm fairly fit, I was walking here about five miles a day.

Reference 2 - 1.95% Coverage

AU3:

For future reference.

RH:

Yeah, okay, so to…

AU3:

To, to help other people who have strokes.

RH:

Okay. Can you expand on that? What, what do you mean by that?

AU3:

Well, if, if I had a stroke, then, because I would, they could, the stroke team would know exactly how to deal with, better, with strokes.

RH:

Okay, so by seeing you and how you developed over six months that would help…

AU3:

Help, yeah.

RH:

…nurses know how to manage…

AU3:

Manage people.

Reference 3 - 0.61% Coverage

AU3:

Well, she was very helpful, *AP1*, wasn't she? She went through with bits and pieces, and medication and things, you know. She took my blood pressure.

[<Files\\Interviews\\AU4.R>](70aaf899-0bdb-47fe-b9dc-bc53fdc6ed3f) - § 1 reference coded [0.46% Coverage]

Reference 1 - 0.46% Coverage

To understand how I’m getting on.

[<Files\\Interviews\\AU5>](2c83a7de-2591-4516-82dc-c6b20e1fddec) - § 2 references coded [1.14% Coverage]

Reference 1 - 0.45% Coverage

I thought it was just to make sure that everything was all right, which I think was, you know, a nice, a nice gesture.

Reference 2 - 0.70% Coverage

I've got the paperwork here and it says, would you like a six month stroke review with one of our specialist nurses discuss any ongoing concerns or difficulties following your stroke?

[<Files\\Interviews\\AU6>](a1e46188-32ea-42de-82dc-e2ee5f63ec0b) - § 1 reference coded [0.51% Coverage]

Reference 1 - 0.51% Coverage

I think it was just to check out a number of aspects of how I've been left with after effects of having the stroke.

[<Files\\Interviews\\BU1>](3d6b994a-2557-4258-93dc-e91fd2300a91) - § 1 reference coded [0.45% Coverage]

Reference 1 - 0.45% Coverage

I think it was just checking up to make sure I'm safe, make sure I'm getting on all right and everything else.

[<Files\\Interviews\\BU3>](4697965c-b754-4519-99dd-03f468e49f76) - § 1 reference coded [0.90% Coverage]

Reference 1 - 0.90% Coverage

Just to find out how I was doing, really. Was I sort of able to look after myself, which I am, and whether I was going out, which I'm not

[<Files\\Interviews\\BU4>](777a5953-4510-44b3-bcdd-03f46d7dbfd9) - § 2 references coded [2.26% Coverage]

Reference 1 - 1.06% Coverage

BU4:

Just to check up, I suppose, on how I was doing. Because you have a six-week coming every day and all of a sudden there's no one there anymore. So, it's a kind of cut-off point.

Reference 2 - 1.20% Coverage

So I think it's just the main thing is, is it just checks up. I think it's good to have a check-up on this degree. So just in case something's wrong in the meantime, you know, referral or something like that.

[<Files\\Interviews\\BU5>](596bbcfc-6e24-4451-8bdd-0d57cb764b65) - § 1 reference coded [0.62% Coverage]

Reference 1 - 0.62% Coverage

To see where I've come from, to where I've got to, what I can keep doing, what medication I've got to stay on and keep taking, how to deal with my life going forward. And really just get over how I felt and how I feel now compared to what I felt in February. To how I feel now

[<Files\\Interviews\\BU6>](908b6c25-06c2-4105-80dd-6d6d748ebae2) - § 3 references coded [1.73% Coverage]

Reference 1 - 0.73% Coverage

BU6:

Just to see if I'm okay or need any help. That's what I understood it. You know, it was a follow-up to see, uh, you know, if how I was getting on and, um, whether or not I needed any help of any sort

Reference 2 - 0.63% Coverage

BU6:

No, not really. They just wanted to know, you know, how I was getting on, really, and, you know, how I was coping, and I told them accordingly. I'm doing all right, really.

Reference 3 - 0.36% Coverage

Did I need any help, as I say? Like I just said, a general well-being check. I would describe it as...

[<Files\\Interviews\\CU1>](c4ae8b9e-52c8-4df6-94dd-623ec555ca51) - § 3 references coded [0.93% Coverage]

Reference 1 - 0.37% Coverage

Yeah, she said that, you know, she was just checking up that I was all right. I could do everything.

Reference 2 - 0.41% Coverage

But we didn't talk about much else. I can't remember anything else. Anyway, she just said had I got any problems.

Reference 3 - 0.15% Coverage

I think she wanted to know if I was safe.

[<Files\\Interviews\\CU2 + husband>](3cf1c6f5-a969-4826-a8dd-623ec6fd33dc) - § 1 reference coded [0.53% Coverage]

Reference 1 - 0.53% Coverage

I think just to come and ask me more questions and just support me as much as anything, I think actually, yes.

[<Files\\Interviews\\CU4>](6d5445c2-2803-4c22-85dd-68639d014623) - § 1 reference coded [0.32% Coverage]

Reference 1 - 0.32% Coverage

Well I suppose I thought it was for me, it was very much for me to find out if I was alright

[<Files\\Observations\\Obs.AP1>](e037026c-6916-4e99-a4dc-bc57b0ac8983) - § 1 reference coded [1.98% Coverage]

Reference 1 - 1.98% Coverage

| Purpose | *Explanation of purpose.*  *Users understanding of purpose.* | To catch-up with how things have been. Check if any worries or concerns.  Patient immediately into problems (fatigue). **Patient seems to be well-informed of the plan for the 6MR via the pre-review information they received. Came with a list of issues they would like to discuss.** |
| --- | --- | --- |

**End of the line**

[<Files\\Interviews\\AU1>](d9bf60e7-662b-41bf-b6dc-a0fab0b5c164) - § 1 reference coded [0.82% Coverage]

Reference 1 - 0.82% Coverage

Was it, was it helpful?

AU1:

No, she said she was going to get back to me. She said she was going to speak to somebody and get back to me and I haven't heard anything yet. So as it happens, no.

[<Files\\Interviews\\AU5>](2c83a7de-2591-4516-82dc-c6b20e1fddec) - § 1 reference coded [0.42% Coverage]

Reference 1 - 0.42% Coverage

So, the six months review was, in a way, superfluous, but nice to know that somebody still carried on, I guess.

[<Files\\Interviews\\AU6>](a1e46188-32ea-42de-82dc-e2ee5f63ec0b) - § 1 reference coded [1.90% Coverage]

Reference 1 - 1.90% Coverage

Do you feel if you hadn't have had the six months review that you would have missed out on anything?

AU6:

I don't think so. I think it was a good way of measuring where I've been, where I was at, and deciding if I needed future help and pointing me in the direction of that. But the six-month review and the stroke team before seemed to be at a point where, okay, it's over to you and your GP now to see how the future lies.

[<Files\\Interviews\\AU7>](e6f5cc0b-9143-4bc3-9edc-e2ee60866caa) - § 1 reference coded [0.66% Coverage]

Reference 1 - 0.66% Coverage

Um, I felt it's for her to find out how I was getting on, but it was an opportunity to… for me to find out any information that I wanted to know, errm concerns.

[<Files\\Interviews\\BU2>](838c03f3-9844-4958-bbdc-e91fd32bb9c3) - § 4 references coded [2.91% Coverage]

Reference 1 - 0.38% Coverage

BU2:

It's too long ago. I can't remember. I really can't remember.

Reference 2 - 1.00% Coverage

You make it sound like she had to get certain information from you.

BU2:

Yes. Yes. She needs a tick, not me.

RH:

Right. So it didn't feel like you got much out of it.

Reference 3 - 0.83% Coverage

BU2:

But I mean, the review I had on the telephone with *BP1* was the end of it. So I mean, I was now perfectly normal to do what I wanted to do.

Reference 4 - 0.70% Coverage

BU2:

No, I think basically it was whatever the system has organised, it was the final thing, yes? Ticking off finals, yes

[<Files\\Interviews\\BU6>](908b6c25-06c2-4105-80dd-6d6d748ebae2) - § 1 reference coded [0.76% Coverage]

Reference 1 - 0.76% Coverage

I suspect, I suspect they've, if you like, signed me off now. I think they have. I've signed me...

They've signed me off as, you know, if I've got any more complaints, see my GP. Yeah. You know, simple as that.

[<Files\\Interviews\\CU2 + husband>](3cf1c6f5-a969-4826-a8dd-623ec6fd33dc) - § 1 reference coded [0.36% Coverage]

Reference 1 - 0.36% Coverage

She really was auditing, if you like, what's going on in those six months

[<Files\\Interviews\\CU4>](6d5445c2-2803-4c22-85dd-68639d014623) - § 1 reference coded [0.65% Coverage]

Reference 1 - 0.65% Coverage

but I also was aware that the NHS has an imperative to follow up things because the structures that are set in place, so it's not just for me it's for everybody to make sure it's working

[<Files\\Observations\\Obs.BP1>](e1f35a7f-46c6-46f4-b7dd-0245f067fc74) - § 1 reference coded [2.84% Coverage]

Reference 1 - 2.84% Coverage

| Purpose | *Explanation of purpose.*  *Users understanding of purpose.* | “How are things?”  ? how much info had beforehand. Patient reported did not receive instructions to check BP prior to review, only that her would be contacted to go through some questions  **At close of 6MR pt asked if *BP1* was happy with the information she had received => ? affirms feeling from interviews that patients feel they are doing the review *for* the provider** |
| --- | --- | --- |

**Safe to proceed**

[<Files\\Interviews\\AU1>](d9bf60e7-662b-41bf-b6dc-a0fab0b5c164) - § 1 reference coded [0.95% Coverage]

Reference 1 - 0.95% Coverage

RH:

Yeah. So I mean, it sounds to me like you had some queries that were still lingering this, this far after your stroke. They've put your mind at ease about some of them or they've helped you work your way through some of that.

[<Files\\Interviews\\AU3>](3770e238-8c1a-46b6-94dc-afe6a72c03c5) - § 1 reference coded [0.57% Coverage]

Reference 1 - 0.57% Coverage

RH:

So I guess it's that you've got that reassurance, you've got a telephone number to call if you need it.

AU3:

That's right, yeah, yeah.

[<Files\\Interviews\\AU4.R>](70aaf899-0bdb-47fe-b9dc-bc53fdc6ed3f) - § 3 references coded [6.60% Coverage]

Reference 1 - 2.79% Coverage

Yes, I felt very reassured afterwards. You need to take responsibility for your own health really don’t you? I think it covered everything. It was nice to have the time to be able to discuss things.

Reference 2 - 1.73% Coverage

No, I think I got to talk about everything I wanted. She gave the time to talk about things and I felt she really listened.

Reference 3 - 2.08% Coverage

I feel reassured. It was nice to be able to talk about all the things that concerned me. I wouldn’t go to the GP with some of these things you know?

[<Files\\Interviews\\AU6>](a1e46188-32ea-42de-82dc-e2ee5f63ec0b) - § 3 references coded [2.19% Coverage]

Reference 1 - 0.68% Coverage

It was reassuring, I think. It's nice to have a third party say, oh, you're doing okay, or you need to concentrate on this or that. So yes, it was useful.

Reference 2 - 0.37% Coverage

So that's where the reassurance came that I'm doing okay as well as can be expected.

Reference 3 - 1.13% Coverage

You talked about almost like getting, I guess it sounds almost like closure from what you're saying.

AU6:

Yeah, I think so. Yeah.

RH:

Yeah.

AU6:

That phase is done now.

RH:

And now it's moving on with the rest of your life.

AU6:

Yeah.

[<Files\\Interviews\\AU7>](e6f5cc0b-9143-4bc3-9edc-e2ee60866caa) - § 4 references coded [3.02% Coverage]

Reference 1 - 0.91% Coverage

And it was lovely because I can't remember how long we'd got, um, or how long we were together, but it wasn't rushed. And that's what I liked. If I had to ask her to explain it again, absolutely all the time in the world.

Reference 2 - 0.71% Coverage

No. It was definitely, it was more personal. Um, you know, she talked about the little dog and it was personal. Whereas on the phone, I wouldn't have remembered who she was.

Reference 3 - 0.18% Coverage

So *AP1* helped me understand all that stuff.

Reference 4 - 1.22% Coverage

six months reviews are important because…

AU7:

Peace of mind. Um, I think that beneficial to people's wellbeing. So mentally and physically probably, but definitely mentally because you're isolated and there was just a lovely group of ladies, but it… yes.

Beneficial to your wellbeing. Yeah.

[<Files\\Interviews\\BP3>](be095d8a-f764-4b5a-84dc-f8ef2b96ce67) - § 1 reference coded [0.45% Coverage]

Reference 1 - 0.45% Coverage

I often ask that. But why are they accepting a six month period if they've got no issues? And again, I think it's that reassurance and that somebody is listening to them and somebody they have that ongoing support.

[<Files\\Interviews\\BU4>](777a5953-4510-44b3-bcdd-03f46d7dbfd9) - § 1 reference coded [0.71% Coverage]

Reference 1 - 0.71% Coverage

But yeah, it was helpful to have the six-week review just to sort of follow up anything that was troubling me, if you like.

[<Files\\Interviews\\CU2 + husband>](3cf1c6f5-a969-4826-a8dd-623ec6fd33dc) - § 1 reference coded [0.47% Coverage]

Reference 1 - 0.47% Coverage

offering any help that she could offer. And that help was giving us pointers to further support.

[<Files\\Interviews\\CU3>](6bbcbf59-fa29-4eca-9bdd-623ec79f1fdc) - § 1 reference coded [1.17% Coverage]

Reference 1 - 1.17% Coverage

Well, I think as well, just a bit of reassurance. I don't, I don't know kind of what I mean by that. Um, but it was nice to have somebody if it was nice to have somebody to come and sit and have the time to sit and listen to the issues that we were having and actually be able to do something about it.

[<Files\\Interviews\\CU4>](6d5445c2-2803-4c22-85dd-68639d014623) - § 1 reference coded [0.76% Coverage]

Reference 1 - 0.76% Coverage

but I felt comforted by it because somebody had remembered me I wasn't alone, and that was good. Especially since I lost my husband, you know it's great to know that there's something there that's looking out for you.

[<Files\\Observations\\Obs.AP2>](41fd7182-554f-4da9-a5dc-bc57b0fa0fa5) - § 1 reference coded [3.01% Coverage]

Reference 1 - 3.01% Coverage

| Context  &  CAS | *Nature of needs and how well are needs met?*  *Adaptations to context i.e. cultural/language, socioeconomic.*  *Carer involvement*  *Interactions between provider and user.* | Patient’s main concern was that his expensive rug had been taken up (because it was a trip hazard)! But had plans to have it put back in place.  Patient would like more balance – concerns over walking ability and risk of falls. He and carer report some near misses.  Attempts made to make data collection (i.e.EQ5D) more natural and part of conversation, but patient struggled giving a straight answer therefore trialed different ways of getting information (i.e. drawing a rating scale).  Supported the identification of current needs to discuss and was providing further perspective in relation to patient needs.  **Easy rapport – lots of laughter.**  **As much time spent telling stories about life as focused on needs. Patient seemed to appreciate this opportunity. Aspects were more like a social visit.** |
| --- | --- | --- |
