# Supplementary material for: Clear tracks or missed connections? A qualitative study exploring how interest-holder perceptions of purpose shape the implementation and experience of the six-month review for stroke survivors
Source: PLoS One. 2025 Dec 11;20(12):e0339038. doi: 10.1371/journal.pone.0339038 (PMC12697933; doi:10.1371/journal.pone.0339038)
Supplement: Attachment — Submitted filename: Response to Reviewers.docx [file pone.0339038.s006.docx]

**Dear Dr Fuller,**

**Thank you for the opportunity to resubmit a revised draft of our manuscript. We appreciate the time and effort spent by yourselves and the reviewers during the review process. We are very grateful for the reviewer’s insightful and encouraging comments and have addressed the suggestions made. Please see a point-by-point response to the reviewers’ comments below.** **Please note, the line numbers highlighting the changes are in reference to the ‘clean’ manuscript.**

Journal Requirements:

When submitting your revision, we need you to address these additional requirements.

1. Please ensure that your manuscript meets PLOS ONE's style requirements, including those for file naming.

Thank you. We have ensured that our manuscript meets all PLOS ONE style requirements, including file naming and document formatting. We have carefully followed the guidelines provided in the journal’s formatting templates for both the main body of the manuscript and the title/author section.

2. We note that your Data Availability Statement is currently as follows: All relevant data are within the manuscript and its Supporting Information files.

Please confirm at this time whether or not your submission contains all raw data required to replicate the results of your study. Authors must share the “minimal data set” for their submission. PLOS defines the minimal data set to consist of the data required to replicate all study findings reported in the article, as well as related metadata and methods ([https://journals.plos.org/plosone/s/data-availability#loc-minimal-data-set-definition](https://gbr01.safelinks.protection.outlook.com/?url=https%3A%2F%2Fjournals.plos.org%2Fplosone%2Fs%2Fdata-availability%23loc-minimal-data-set-definition&data=05%7C02%7Crichard.holmes8%40nhs.net%7C67ea7ee635ef47a3e75208de1bd034c9%7C37c354b285b047f5b22207b48d774ee3%7C0%7C1%7C638978778262962080%7CUnknown%7CTWFpbGZsb3d8eyJFbXB0eU1hcGkiOnRydWUsIlYiOiIwLjAuMDAwMCIsIlAiOiJXaW4zMiIsIkFOIjoiTWFpbCIsIldUIjoyfQ%3D%3D%7C0%7C%7C%7C&sdata=uolhF8TbqoANOClg9%2B94zseLCor80%2B8G2cppPE%2BHSKY%3D&reserved=0)

Authors do not need to submit their entire data set if only a portion of the data was used in the reported study.

If your submission does not contain these data, please either upload them as Supporting Information files or deposit them to a stable, public repository and provide us with the relevant URLs, DOIs, or accession numbers. For a list of recommended repositories, please see [https://journals.plos.org/plosone/s/recommended-repositories](https://gbr01.safelinks.protection.outlook.com/?url=https%3A%2F%2Fjournals.plos.org%2Fplosone%2Fs%2Frecommended-repositories&data=05%7C02%7Crichard.holmes8%40nhs.net%7C67ea7ee635ef47a3e75208de1bd034c9%7C37c354b285b047f5b22207b48d774ee3%7C0%7C1%7C638978778262974694%7CUnknown%7CTWFpbGZsb3d8eyJFbXB0eU1hcGkiOnRydWUsIlYiOiIwLjAuMDAwMCIsIlAiOiJXaW4zMiIsIkFOIjoiTWFpbCIsIldUIjoyfQ%3D%3D%7C0%7C%7C%7C&sdata=q9YrjlxrPdK3eovXSOpjPgIrV7i195%2FEzGDtMZ%2Bqnuk%3D&reserved=0).

If there are ethical or legal restrictions on sharing a de-identified data set, please explain them in detail (e.g., data contain potentially sensitive information, data are owned by a third-party organization, etc.) and who has imposed them (e.g., an ethics committee). Please also provide contact information for a data access committee, ethics committee, or other institutional body to which data requests may be sent. If data are owned by a third party, please indicate how others may request data access.

We have added an additional document in the supporting information **(S3 File)**. This includes all the anonymised coded data used in the thematic analysis for this study. We trust that this sufficiently meets the ‘minimal data set’ requirement.

3. If the reviewer comments include a recommendation to cite specific previously published works, please review and evaluate these publications to determine whether they are relevant and should be cited. There is no requirement to cite these works unless the editor has indicated otherwise.

Thank you. Reviewer 1 highlights a useful paper to aid terminology. We have gratefully taken their suggestion on this point but did not feel the need to reference the published work.

**Reviewer #1**: Thank you for the opportunity to review this clearly written and interesting article. I hope the comments below are of use in further improving this manuscript.

Thank you for taking the time to review our manuscript, it is much appreciated.

Abstract: It’s unclear from the abstract methods section that this submission only reports on one element of data collection i.e. the interviews. I would suggest removing reference to the other methods of data collection within the abstract.

Thank you. We acknowledge that this could have been clearer. Our second reviewer raised similar concerns regarding how we have described the approach used. This manuscript reports on the thematic analysis of *purpose* which was one aspect of consideration within a wider project. To clarify, all 3 methods of data collection were used in the thematic analysis (albeit with the vast majority of data and insights coming from the interviews). We have altered the Abstract (lines 30-31) and the design section of the Methods (lines 110- 113) to clarify our approach and the relation to the wider multiple case study.

You have used the term stakeholders within this submission. You may wish to use different terminology for a global audience such as interest holders. Please refer to the following reference for more information: Akl, EA, Khabsa, J, Petkovic, J, Magwood, O, Lytvyn, L, Motilall, A, Campbell, P, Todhunter-Brown, A, Schünemann, HJ, Welch, V, Tugwell, P & Concannon, TW 2024, '“Interest-holders”: A new term to replace “stakeholders” in the context of health research and policy', Cochrane Evidence Synthesis and Methods, vol. 2, no. 11, e70007. <https://doi.org/10.1002/cesm.70007>

We are very grateful to our reviewer for providing this reference which highlights the culturally-sensitive nature of this term. We have followed this suggestion and changed stakeholders to interest-holders throughout the manuscript. However, we note that the editorial team at PLOS One may have a particular journal convention/style for such terms, therefore we will be guided by their decision.

Methods: Given the role of the lead researcher in 6MR development – does this mean you knew some of the staff prior to the interviews? Information on any relationships with participants would be helpful here as per the COREQ checklist.

Thank you for raising this and apologies this was not clear. Our second reviewer also raised a similar point. We can confirm that none of the authors knew any of the staff prior to the interviews. Confusion may have arisen through the statement: ‘he has also provided clinical leadership for the commissioning of 6MR services’. This statement reflects a role as a clinical expert involved in the commissioning of 6MR services rather than leading a clinical team. We have edited the text to reflect this and hope it clarifies (lines 123-125 and 131-132).

More detail on how the reflexive thematic analysis was undertaken would be helpful e.g. did you undertake line by line coding to generate initial themes which were then refined?

Yes, this was the process used, and we have added text to clarify this in the Methods section (lines 189-194.

It’s unclear what a collaborative reflexive approach was- can you please clarify?

Thank you. The sentence containing ‘collaborative reflexive approach’ is merely a repetition of what is described in the following 2 sentences (lines 194-196), we have therefore removed this sentence to avoid confusion.

It would be helpful to know how much time was spent on 6MR appointments and did this vary throughout different services to provide further context.

Thank you for your interest in this nuanced aspect of the 6MR. Time spent in the review varied considerably *within* services more so than *between* services. The reasons for this are complex and are heavily influenced by context. We acknowledge this important point and discuss this in a separate (forthcoming) article in which these variations are explored in detail. We therefore felt it would be beyond the scope of the current paper, which focuses specifically on perceptions of purpose.

P15 abbreviations are used within the quote – it would be helpful to explain what these are for readers.

Thank you, quotes were added verbatim therefore we have provided a short explanation within the quote (lines 315-317).

Discussion: It is noted that all your participants were white, I am pleased to see that this is acknowledged within your limitations but given that other ethnicities can have a higher prevalence of stroke and are less likely to attend 6MR perhaps a recommendation that further work should address this is needed?

Thank you for this suggestion. Text has been added (lines 525-527).

Thank you for providing a summary table – this is helpful for readers. I also enjoyed your use of themes aligned to train travel. Finally, well done for including people with aphasia and cognitive deficits within this research.

Thank you again for your kind comments.

**Reviewer #2**: ONE-D-25-27773 Clear tracks and missed connections: A qualitative study exploring how stakeholder perceptions of purpose shape the implementation and experience of the six-month review for stroke survivors. S

A pleasure to read but it will be even better with a little more work, and demonstrate clearly how the conclusions were reached. The main weakness is stating that it was a case study design but not demonstrating this in the results or discussion (unless I am misunderstanding your use of the term).

Thank you for highlighting this and apologies for the lack of clarity on this point. We think clarifying this point will help to address a number of the concerns below.

While the broader BE MoRe project used a multiple case study approach to capture contextual variation between sites, this paper reports a sub-analysis of the data exploring differing views on the purpose of the 6MR using a reflexive thematic analysis of the interview, observational, and documentary data across all sites. Rather than comparing sites, our analysis focused on identifying and comparing themes between interest-holder groups. A further layer of site-level comparison was considered but deemed impractical given the small subgroup sizes (in some instances 1-2 participants).

In the abstract (lines 30-31) and methods (lines 110- 113), we have clarified that while the data was taken from a multiple case study, the data analysis for this aspect of the study considered the dataset as a whole, split into participant groups and analysed using a reflexive thematic approach.

Introduction: covers the main points but might be worth mentioning that 6MR is part of process than includes review at 6weeks, 6 months and yearly, but that timeframe of reviews was arbitrary. You could then pick this up in the discussion.

We thank our reviewer for this point. We share the reviewer’s views on the importance of these reviews; however, in practice, the 3 different review points are often very different in structure and function (if they happen at all) and delivered by different services. We have therefore decided not to include this detail in the current paper so as not to detract from the central focus of the 6MR.

Line 79-80 mentions unmet needs (McKevitt et al 2011) as rationale to support the 6MR but it would be worth adding a sentence to qualify if this is still the case (or more likely, that need is greater in current economic/healthcare climate) and any recent papers to support this.
Thank you for this helpful point. The McKevitt paper is referenced to provide historical context for the inclusion of the 6MR in recommendations. Our intention was to link this to the more recent reference that follows (Zawawi et al, 2020) that highlights the persistence (and evolving range) of unmet needs, supporting the ongoing relevance of this issue. We have clarified this in the text to make the link between historical and recent evidence more explicit (line 80).

Method:
Study Design, p5-6: states case study design (please clarify what type, there are several), tools (interview/obs/documentary analysis) and paradigm (pragmatic) but the rationale for all these, and how they worked together, needs stronger/clearer justification.

Again we apologise for the lack of clarity in our description of the study. We feel this is now clearer with the edits made in response to the points below.

Lines 117-9 are non-sequiturs.
Thank you for highlighting this. We have added a sentence to improve the flow in this paragraph. Specifically, we have added a sentence to highlight that the pragmatic perspective emphasises generating knowledge that can be applied to real-world problems, which naturally leads to insights that are sensitive to context and can inform practice and policy (lines 118-120).

While it states that it is a multiple case study design, this is not clear in the text – in line 132 it states ‘3 stakeholder groups linked to three 6MR services’ but this is not described (who, how were the sites recruited, why defining features).
Thank you for highlighting. We have moved the final paragraph in the ‘Sample’ section to the beginning and added more detail to clarify. We hope this provides a more logical progression describing how the sites were recruited from the initial quantitative phase of the project.

Please add a description of the case study sites – this need to be added to include how sites were selected (and it’s ok to say it was whoever you could get), how they differ, the wider context, and a description of the model of 6MR for each site (e.g. who carries it out, where, how, whether they use a standardised tool or their own form etc). This information could be tabulated to save words. I’m assuming (from lines 120-125) that the author works in one of the sites but this isn’t clear.

Thank you for this comment. As above, we have added more detail to show how sites were selected. We have also included a table providing more detail of each site than is already in the text (**S1 Table**). However, since this paper does not present the full case-level analysis, we have limited site descriptions to essential contextual detail; a fuller account will be provided in a forthcoming paper.

What was the unit of analysis for the case study site (if you’re using Yin)?

As above, we hope to have clarified that this paper is not reporting on the multiple case study approach of the wider study. For interest and context, in the wider study, we were guided by Merriam’s case study approach rather than Yin’s.

Sample size: How many interviews did you intend to carry out for each group, and at each site?
What was your approach to data saturation – it’s ok to say you did what you could in the time you had but be explicit.
Thank you for this point. We set out with an estimated intention of recruiting 2-3 providers, 2-3 influencers, and 6-8 users at each site. However, this was purely used as an estimate during the planning stages and we were aware that the actual recruitment numbers would be guided by the nuances for each case (e.g. if a service only had a single provider we would be limited to one). Using Braun and Clarke’s reflexive thematic analysis approach, the final sample size was a reflexive decision based on when sufficient data was gathered to capture the nuances of each case. Text to this effect has been added to the manuscript (lines 145-149)..

Recruitment: it’s not clear how services were approached and then how service users were recruited – lines 143-4 only state the end point, that staff/research invited them, but this misses how you got to that point.
Thank you. We have included detail to describe how the services were selected and recruited into the study (lines 135-139).

Observations: unclear what/who/how you observed each of the formats (line 164), including phone calls. Nursing/residential homes are mentioned (p8, line 165) but not mentioned elsewhere – including data specific to NH/RHs would enhance the results.
Thank you. We have included additional detail regarding observations (173-174) and have added the data collection sheet used for all observations to the supplementary document **(S2 File).**

The mention of nursing homes is intended to show the breadth of observations undertaken. Specific analysis related to nursing homes was not undertaken within the remit of this aspect of the project.

Topic guides: worth including a summary of key topics in the text, and the full versions keep as supplementary.

Thank you for this suggestion. We have added text to summarise the key relevant topics, noting that the topic guides were different for each interest-holder group (lines 167-170).

Data analysis, p8: please add further information on:
- How you analysed observational data (within and across sites)
- How you analysed documents (within and across sites)
- How you carried out thematic analysis –more detail e.g. the nuances of how you compared across stakeholder groups – and between sites, which is absent.
- How you synthesised the data within & across the sites - obs, documents, interviews (staff, influences & patients/carers)
- How you used CICI framework

With further information on the above, it will be clearer how you have addressed rigour/trustworthiness.

Thank you for your consideration of these details. We hope our clarification that this aspect of the project used thematic analysis will explain our approach to data analysis. The observational and documentary data were coded in the same way as interview data. All data were handled in the same way. We have clarified within the manuscript (lines 189-190) that observational and documentary data were coded alongside interview data within a single reflexive thematic framework, thereby ensuring analytic consistency across sources.

Thank you for considering our use of CICI. While it was not applied directly in the data collection or analysis of *purpose* in the context of this aspect of the study, its use within the wider project informed the interpretation of our findings. On reflection, we agree it received disproportionate attention in the current paper; we have therefore removed mention of CICI from the ‘Data collection’ and ‘Strengths’ section, and removed from the reference list.

Results
As above, it states that it is a multiple case study design but the analysis does not reflect this which makes it hard to comment on the results. While quotes are attributed to each site it is not clear how the sites differed and what these differences meant e.g. in terms of patient experience or outcomes. For example:
- p11 mentions that the 6MR shouldn’t be a checklist approach but it’s unclear how the reviews were carried out and what, if any (checklist style) tools were used.
- Bottom of p11 mentions the importance of dedicated time with the stroke survivor, but the reader does not know if the person carrying out the 6MR has met them before during the rehab phase, or carried out a 6 week review, or is going in cold. Nor do we know if patients/carers were able to contact the Reviewer afterwards with any queries – some clinicians provide a phone number, others don’t, but this is relevant & would come under descriptions of the 3 sites.

Thank you for your comments. We have added a table (**S1 Table**) as suggested earlier that provides further detail about the sites. However, we want to highlight that in this analysis the focus is on the *perceptions* of interest-holders specifically in relation to *purpose*; within this paper we are not linking these perceptions to specific aspects of delivery such as data collection tools or follow-up. The analysis of cases, including how the context of each case influences outcomes, will be discussed in a forthcoming paper.

Once you have addressed the points under data analysis, then briefly present the key findings – you could cut by observations, documentary analysis, and interviews and/or by site but for case study design you need to be clear about differences/similarities between sites and this is lacking. You can integrate documentary & obs with interview data but be explicit what is from which category. You could even include notes from your field log.

Thank you for this suggestion. We hope this comment is resolved through clarification we have provided regarding the analysis, therefore we have maintained the current structure of the results split by different interest-holder groups.

Some of the sub-headings sound good but lack clarity/meaning – it might be worth expanding the descriptor e.g. ‘charting the course of……’. Under this theme it is unclear what data was collected, which would be useful to state under the theme or under the site descriptions.

Thank you for this suggestion. However, we feel this falls outside of the remit of this paper. We do not aim to present what data the influencers were collecting, only that they perceived the purpose of the 6MR was to collect data.

I’m not convinced that Table 3 is useful – it’s not clear the interconnections between themes, as Fig 1 aims to represent. See when you’ve reviewed the results – the Table might be better as supplementary information.

Thank you for your suggestion. We have decided to keep the summary within the main text. We are mindful that 7 themes across 3 different interest-holder groups is a lot to keep track of and feel that some readers may find the table a helpful reference point as per Reviewer 1’s comment above.

Discussion
Overall, good points but needs to be further developed/refined, and ensure that all statement are backed up by what’s been presented in the results (see below).
Line 447 – mentions wide variation in delivery of 6MR in UK – as above, this needs to be mentioned in the introduction and lead into your choice of case study sites / types of 6MR.
Thank you. The second paragraph in the introduction mentions the quantitative stage of this project which highlighted wide variation. We have added a line to link this to the selection of sites in the methods (lines 135-137).

Figure 1 makes good sense but Figure 2 needs to be further developed and explained/justified in the text. Currently, it doesn’t add to the text. In the same paragraph (lines 456-459), its proposed that systems with high value on the 6MR take one approach versus those that see it as low priority and take a different direction – but we can’t see that in the results, and you need to back it up – hence why it’s important to describe the three settings and the differences between them.

Thank you for highlighting this. Here we attempt to suggest a potential hypothesis to explain the variation seen in practice and how this relates to providers’ operationalisation of their ideations of purpose. However, we acknowledge that the example we have provided brings in knowledge from our wider study and feels out of place here. We have therefore adjusted the text to be more conceptual (lines 474-477). We have also adjusted the text within the discussion to clarify the interactions in figure 2 (lines 470-474) and we have added a legend to figure 2.

Study limitations – well covered.

Thank you.

Conclusion – this brings in programme theory which hasn’t been mentioned before and seems somewhat out of place (given it’s not a realist evaluation). The final sentence about future research would benefit from refining.

Thank you. Earlier in the manuscript we discuss the ‘theoretical underpinnings’ but have changed this to programme theory to maintain consistency (line 110). The development of programme theory is the overarching aim of the wider project. We also note that, while programme theory is often associated with realist evaluation, it is not exclusively linked to that methodology. In this study, we use programme theory in its broader sense to articulate how the 6MR is understood to work based on interest-holders’ perspectives without implying a full realist evaluation.

**Once again, we wish to express our gratitude for taking the time to undertake this thorough peer-review process. We are grateful for the reviewer’s insightful comments in relation to this important aspect of stroke care, and we are confident that their input has led to greater clarity within our manuscript.**

**Sincerely,**

**Rich Holmes**

**Corresponding author**

**Physiotherapy Department, St Richard’s Hospital,**

**Chichester, UK, PO19 6SE**

**Email: richard.holmes8@nhs.net**
